# Histone deacetylase inhibitor-induced cancer stem cells exhibit high pentose phosphate pathway metabolism

**DOI:** 10.18632/oncotarget.8631

**Published:** 2016-04-07

**Authors:** Bisrat G. Debeb, Lara Lacerda, Richard Larson, Adam R. Wolfe, Savitri Krishnamurthy, James M. Reuben, Naoto T. Ueno, Michael Gilcrease, Wendy A. Woodward

**Affiliations:** ^1^ Department of Radiation Oncology, The University of Texas MD Anderson Cancer Center, Houston, TX 77030, USA; ^2^ Department of Pathology, The University of Texas MD Anderson Cancer Center, Houston, TX 77030, USA; ^3^ Department of Hematopathology, The University of Texas MD Anderson Cancer Center, Houston, TX 77030, USA; ^4^ Department of Breast Medical Oncology, The University of Texas MD Anderson Cancer Center, Houston, TX 77030, USA; ^5^ MD Anderson Morgan Welch Inflammatory Breast Cancer Research Program and Clinic, Houston, TX 77030, USA

**Keywords:** HDAC inhibitors, cancer stem cells, pentose phosphate pathway, G6PD

## Abstract

**Purpose:**

We recently demonstrated that histone deacetylase (HDAC) inhibitors can “reprogram” differentiated triple-negative breast cancer cells to become quiescent stem-like cancer cells. We hypothesized that the metabolic state of such cells differs from that of their differentiated progeny.

**Results:**

In untreated cells, glucose uptake was higher in ALDH^+^ cells than in ALDH^−^ cells (*p* = 0.01) but lactate production was not different; treating ALDH^−^ or ALDH^+^ cells with VA or SAHA similarly increased glucose uptake without changing lactate production but upregulated G6PD, a rate-limiting enzyme in pentose phosphate pathway metabolism. NADPH production was higher in HDAC inhibitor-treated stem-like cells than in vehicle-treated cells (*p* < 0.05). Two G6PD inhibitors, 6-aminonicotinamide and dehydroepiandrosterone, decreased mammosphere formation efficiency and ALDH activity and 6-aminonicotinamide reduced the VA-induced increase in ALDH^+^ cells. Finally, patients expressing high G6PD mRNA had significantly worse overall survival (*p* < 0.001), and patients with high G6PD protein showed a similar trend towards worse disease-specific survival (*p* = 0.06).

**Methods:**

Glucose consumption, lactate and NADPH production, and reactive oxygen species generation were compared in aldehyde dehydrogenase (ALDH)-positive and –negative cells in the presence or absence of the HDAC inhibitors valproic acid (VA) or suberoylanilide hydroxamic acid (SAHA). Glucose-6-phosphate dehydrogenase (G6PD) expression was evaluated in a tissue microarray from 94 patients with node-positive invasive breast carcinoma and in two publically available databases and correlated with overall survival.

**Conclusions:**

Energy metabolism in HDAC inhibitor-induced stem-like cancer cells differed sharply from that of differentiated cell types. HDAC inhibitor-induced dedifferentiation promoted metabolic reprogramming into the pentose phosphate pathway, which is targeted effectively by G6PD inhibition. These findings highlight a potential dual-therapy approach to targeting bulk differentiated cells with HDAC inhibitors and CSCs with G6PD inhibitors.

## INTRODUCTION

The identification of cancer stem cells in solid tumors has opened a new avenue for understanding cancer biology and developing targeted therapies. Cancer stem cells (CSCs), also called tumor-initiating cells, are a small population of cancer cells with stem cell properties, including the capacity to self-renew and the ability to differentiate into multiple cell types. Putative human breast cancer stem cells have been identified based on their expression of specific cell surface markers such as CD44^hi^/CD24^lo^ [1] and aldehyde dehydrogenase (ALDH) activity [2] and can be enriched *in vitro* as non-adherent spheres (mammospheres) [3].

CSCs are thought to be responsible for recurrence after clinical remission because they are more resistant to therapy. We and others have shown evidence that CSCs from breast tumor cells are resistant to radiation [4–6]. Chemotherapy has also been shown to increase the percentage of the cell population with CD44^hi^CD24^lo^ surface markers [7, 8]. Moreover, several studies have implicated CSCs in breast cancer metastasis [9, 10]. Because of the importance of CSCs in therapy resistance and metastasis, significant efforts are being undertaken to identify new therapeutic approaches to target this type of cells. Given the importance of metabolic reprogramming in tumorigenesis and therapy resistance, the metabolic state of CSCs could be exploited to design new strategies to target tumor growth and recurrence.

The Warburg effect (the dependence of most cancer cells on glycolysis to produce energy) is thought to be a universal phenomenon in all cancer cells. However, it is plausible that differences in metabolic state exist in heterogeneous tumors composed of CSCs and differentiated cells. Literature on the metabolic state of CSCs and their differentiated progeny is contradictory. Some studies comparing the metabolic state of glioma, leukemia, and breast CSCs with the differentiated cell population demonstrated that CSCs rely more on oxidative phosphorylation than differentiated cells [11–13]. However, others have shown that CSCs are more dependent on anaerobic glucose metabolism [14–16]. We recently demonstrated that histone deacetylase (HDAC) inhibitors promote the expansion of the CSC subpopulation by reprogramming differentiated cancer cells into stem-like cells [17]. In the present study we investigated whether these stem-like breast cancer cells are metabolically different from the differentiated cancer cells. We report that HDAC inhibitor-induced stem-like cancer cells have an enhanced pentose phosphate pathway (PPP) metabolism.

## RESULTS

**Figure 1:**
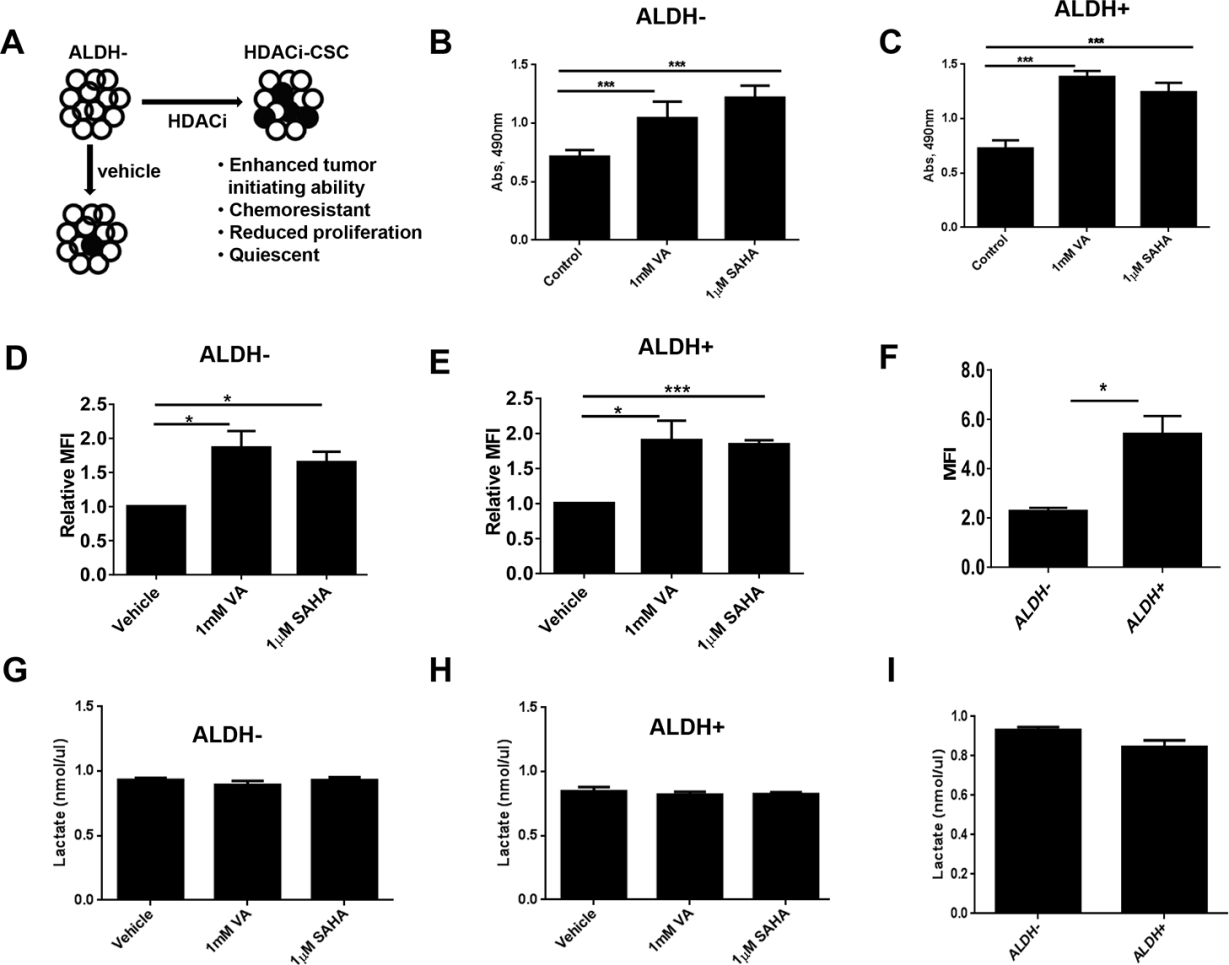
Metabolic activity of HDACi-CSCs and differentiated breast cancer cells (**A**) The histone deacetylase (HDAC) inhibitors valproic acid (VA) and suberoylanilide hydroxamic acid (SAHA) reprogram differentiated cancer cells into chemoresistant, quiescent cancer stem-like cells (designated here HDACi-CSCs). ○ALDH^−^; ●ALDH^+^. (**B** and **C**) An MTT assay showed that treating ALDH^−^ (B) and ALDH^+^ (C) SUM159 cells with HDAC inhibitors for 7 days significantly increased their metabolic activity compared with vehicle-treated cells. (**D** and **E**) Flow cytometric analysis of glucose uptake using the glucose analog 2-[N-(7-nitrobenz-2-oxa-1,3-diaxol-4-yl) amino]-2-deoxyglucose (2-NBDG) in ALDH^−^ (D) and ALDH^+^ (E) cells treated with HDAC inhibitors versus vehicle-treated controls. Glucose uptake of HDACi-CSCs was significantly higher than that of differentiated cells. (**F**) Glucose uptake significantly higher in untreated ALDH^+^ versus ALDH^−^ cells. (**G** and **H**) Lactate production, analyzed with a lactate colorimetric assay, revealed no differences in lactate levels in ALDH^−^ (G) or ALDH^+^ (H) cells treated with HDAC inhibitors versus vehicle-treated controls. (**I**) No difference in lactate production was observed in untreated ALDH^+^ versus ALDH^−^ cells.

We previously found that HDAC inhibitors can reprogram differentiated triple-negative breast cancer cells to become “induced” tumorigenic, chemoresistant stem-like cells [17] (HDACi-CSCs, Figure 1A). Unsorted cells treated with HDAC inhibitors also showed significantly higher ALDH^+^ subpopulation versus vehicle-treated cells (Supplementary Figure 1A). Moreover, both ALDH^−^ and ALDH^+^ cells treated with HDAC inhibitors had significantly lower proliferation rates and were quiescent ([17] and Supplementary Figure 1B, 1C). However, an MTT assay, widely used to assess the proliferation rate of cancer cells based on metabolic activity, unexpectedly showed that HDACi-CSCs from both SUM159 ALDH^−^ and ALDH^+^ parental cells had much higher activity than did vehicle-treated controls (*p* < 0.0001, Figure 1B, 1C). These observations led us to further examine potential metabolic differences in HDACi-CSCs versus differentiated breast cancer cells. We first examined the glucose uptake of HDACi-CSCs from ALDH^−^ and ALDH^+^ cells pretreated with 1 mM valproic acid (VA) or 1 μM suberoylanilide hydroxamic acid (SAHA) for 7 days by using fluorescent 2-deoxyglucose analog 2-[N-(7-nitrobenz-2-oxa-1,3-diaxol-4-yl) amino]-2-deoxyglucose (2-NBDG). We found that HDACi-CSCs had significantly higher 2-NBDG uptake than did un-induced cells in both ALDH^−^ and ALDH^+^ subpopulations (ALDH^−^: vehicle vs 1 mM VA, *p* = 0.03; vehicle vs 1 μM SAHA, *p* = 0.01; ALDH^+^: vehicle vs 1 mM VA, *p* = 0.03; vehicle vs 1 μM SAHA, *p* = 0.0002, Figure 1D, 1E). Moreover, in the untreated parental subpopulations, glucose uptake in the stem-like ALDH^+^ population was significantly higher than the differentiated ALDH^−^ population (5.40 ± 0.73 vs. 2.27 ± 0.14, *p* = 0.01, Figure 1F). We next examined the byproduct of anaerobic glycolysis, lactate, in the HDACi-CSCs from ALDH^−^ and ALDH^+^ populations and differentiated subpopulations and found that lactate production was not different in the pretreated cells versus their controls and in the stem-like cells versus committed cancer cells (*p* > 0.05, Figure 1G, 1H, 1I).

**Figure 2:**
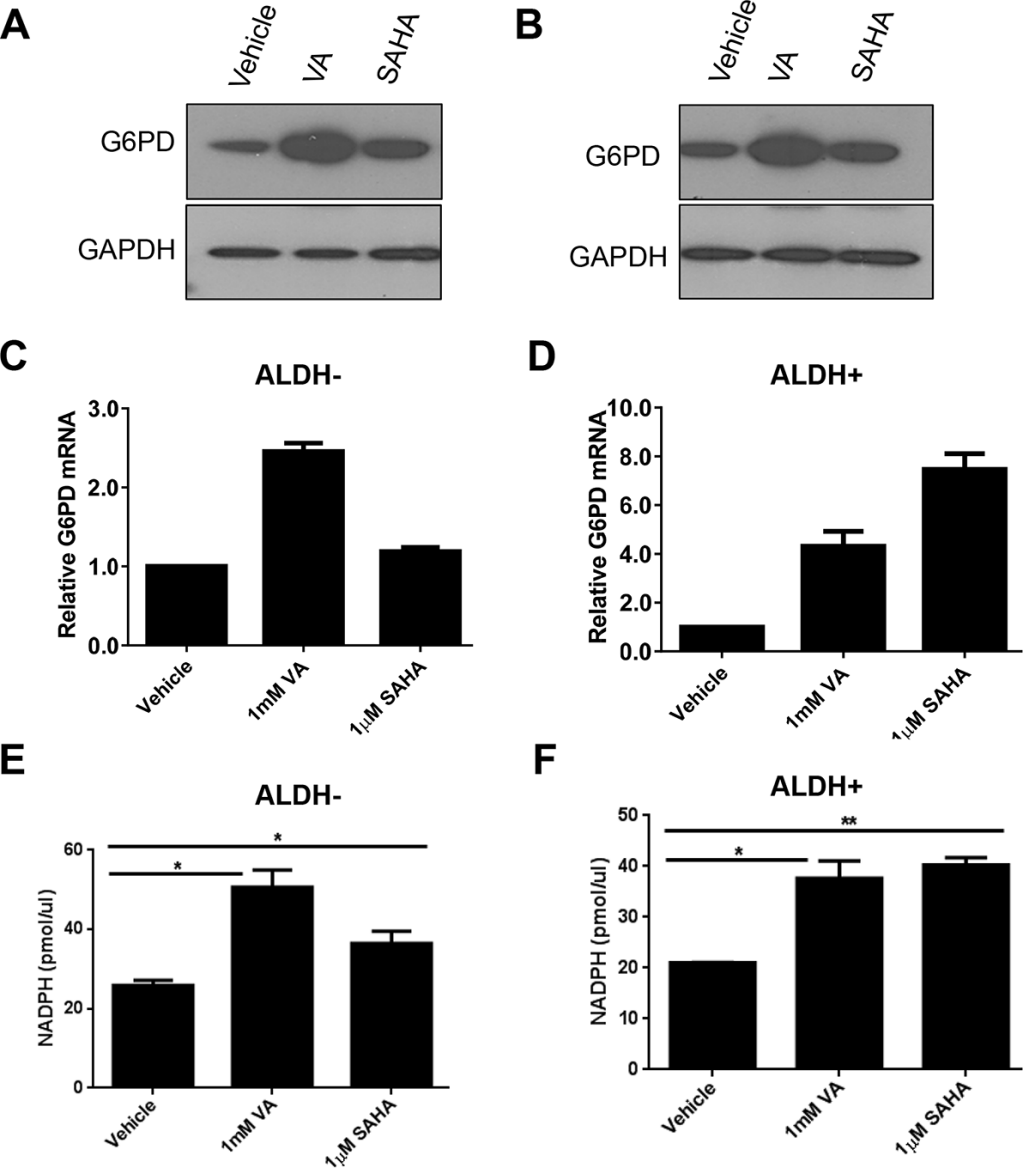
Enhanced pentose phosphate pathway (PPP) metabolism in HDACi-CSCs (**A** and **B**) Elevated levels of glucose-6-phosphate dehydrogenase (G6PD) protein, a rate-limiting enzyme in the PPP, in ALDH^−^ (A) and ALDH^+^ (B) subpopulations treated with VA or SAHA compared with vehicle-treated controls. (**C** and **D**) Increased G6PD mRNA in ALDH^−^ (C) and ALDH^+^ (D) cells treated with VA or SAHA compared with vehicle-treated cells. (**E** and **F**) NADPH production was higher in ALDH^−^ (E) and ALDH^+^ (F) cells treated with VA or SAHA compared with vehicle-treated cells.

**Figure 3:**
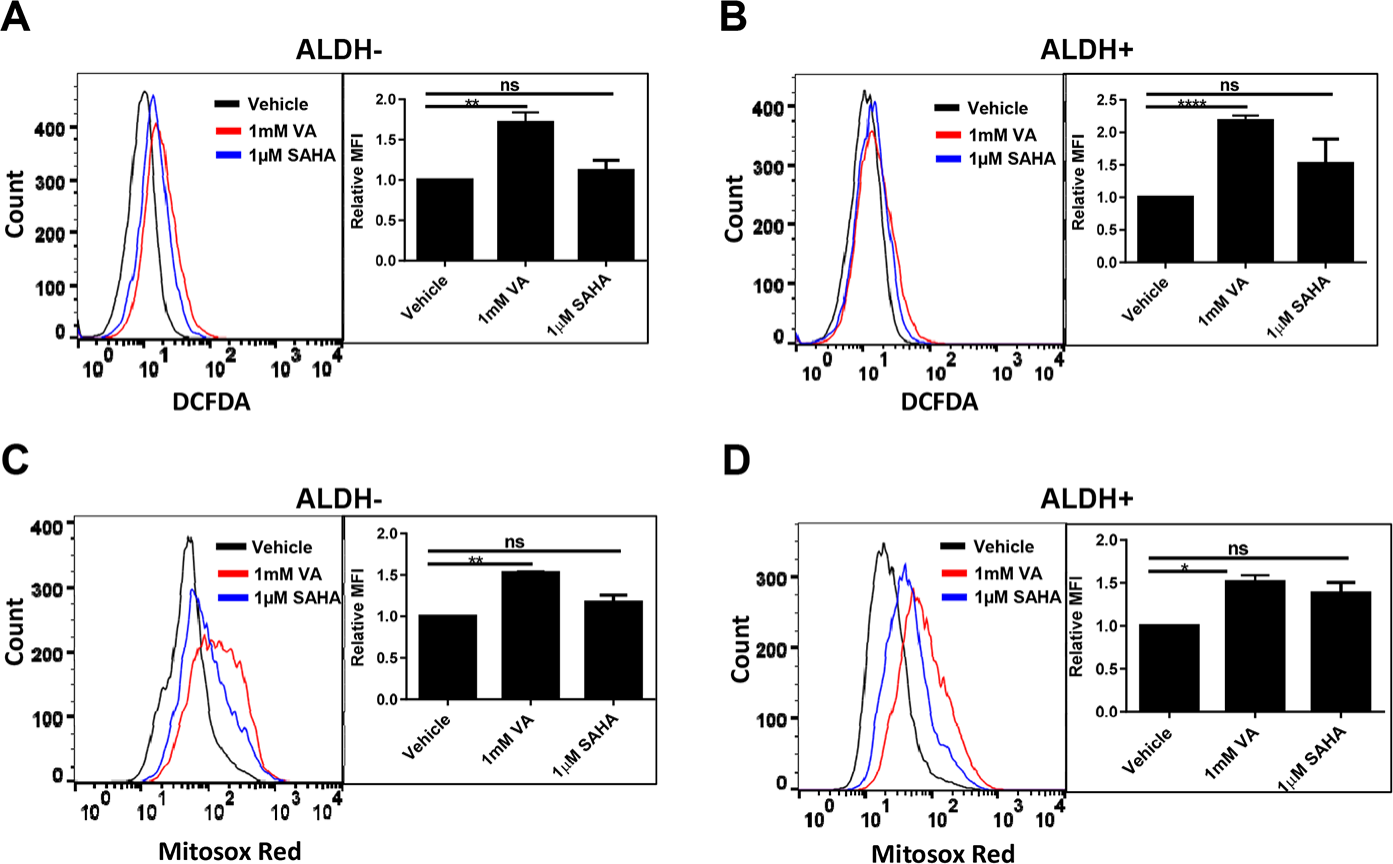
ROS levels in in HDACi-CSCs and differentiated cancer cells (**A** and **B**) Flow cytometric analysis of ROS with 2′-7′-dichlorofluorescein diacetate (DCF-DA). ROS concentrations were higher in ALDH^−^ (A) and ALDH^+^ (B) cells treated with VA versus vehicle-treated controls. (**C** and **D**) Flow cytometric analysis of mitochondrial superoxide with MitoSOX Red. Superoxide levels were higher in VA-induced HDACi-CSCs from ALDH^−^ (C) and ALDH^+^ (D) cells than in vehicle-treated differentiated cells. Representative overlaying merged graphs are shown with bar graphs summarizing quantification of the DCFDA and Mitosox-red data from several experiments.

Cancer cells consume large quantities of glucose and primarily use glycolysis for ATP production, even in the presence of adequate oxygen (Warburg effect). These cells metabolize glucose by both the glycolytic pathway and the PPP. In the glycolytic pathway, higher levels of glucose uptake result in the generation of ATP and pyruvate, which is directed away from the mitochondria to generate lactate through the action of lactate dehydrogenases. In the PPP, which branches from glycolysis at the first committed step of glucose metabolism, the glycolytic intermediates are diverted to provide anabolic precursors for nucleotide synthesis and to maintain cellular antioxidant defenses of cancer cells, supporting their rapid growth and proliferation [18]. On the basis of our observing an increase in glucose consumption with no change in lactate production in the induced cancer stem-like population (Figure 1), we hypothesized that HDACi-CSCs have an enhanced PPP metabolism compared with the differentiated subpopulation. We first examined the protein levels of glucose-6-phosphate dehydrogenase (G6PD), the tightly regulated, rate-limiting enzyme in the PPP, and found that they were elevated in ALDH^−^ or ALDH^+^ subpopulations treated with VA or SAHA relative to vehicle-treated controls (Figure 2A, 2B). HDAC inhibitors, particularly VA, also increased G6PD mRNA expression in ALDH^−^ or ALDH^+^ cells (Figure 2C, 2D). The PPP has a significant role in the production of cellular NADPH (nicotinamide adenine dinucleotide phosphate, reduced), which is required for the synthesis of fatty acids and for detoxification of intracellular reactive oxygen species (ROS). As expected, NADPH levels were significantly higher in ALDH^−^ or ALDH^+^ cells treated with VA or SAHA than in the vehicle-treated cells (ALDH^−^: vehicle vs 1 mM VA, 25.9 ± 0.9 vs 50.6 ± 3.1, *p* = 0.02; vehicle vs 1 μM SAHA, 25.9 ± 0.9 vs 36.5 ± 2.2, *p* = 0.04; ALDH^+^: vehicle vs 1 mM VA, 21.0 ± 0.03 vs 37.7 ± 2.4, *p* = 0.02; vehicle vs 1 μM SAHA, 21.0 ± 0.03 vs 40.3 ± 1.0, *p* = 0.003, Figure 2E, 2F), supporting enhanced PPP activity in HDACi-CSCs versus in differentiated cells. To compare ROS levels between HDACi-CSCs and differentiated cells, we measured intracellular concentrations of pro-oxidants by using 2′-7′-dichlorofluorescein diacetate (DCF-DA) staining with flow cytometry. HDACi-CSCs generated from VA treatment had significantly higher concentrations of ROS than did the vehicle-treated differentiated subpopulation (ALDH^−^: vehicle vs 1 mM VA, *p* = 0.001; ALDH^+^: vehicle vs 1 mM VA, *p* = 0.0001, Figure 3A, 3B). Similarly, analysis of these two populations with MitoSOX Red, a highly selective detection method for mitochondrial superoxide, revealed higher superoxide levels in the VA-induced HDACi-CSCs population than in vehicle-treated differentiated population (ALDH^−^: vehicle vs 1 mM VA, *p* = 0.0003; ALDH^+^: vehicle vs 1 mM VA, *p* = 0.02, Figure 3C, 3D). We also found lower levels of the ROS scavenger SOD2 in reprogrammed stem cell-like cells vs. differentiated cancer cells (Supplementary Figure 2).

**Figure 4:**
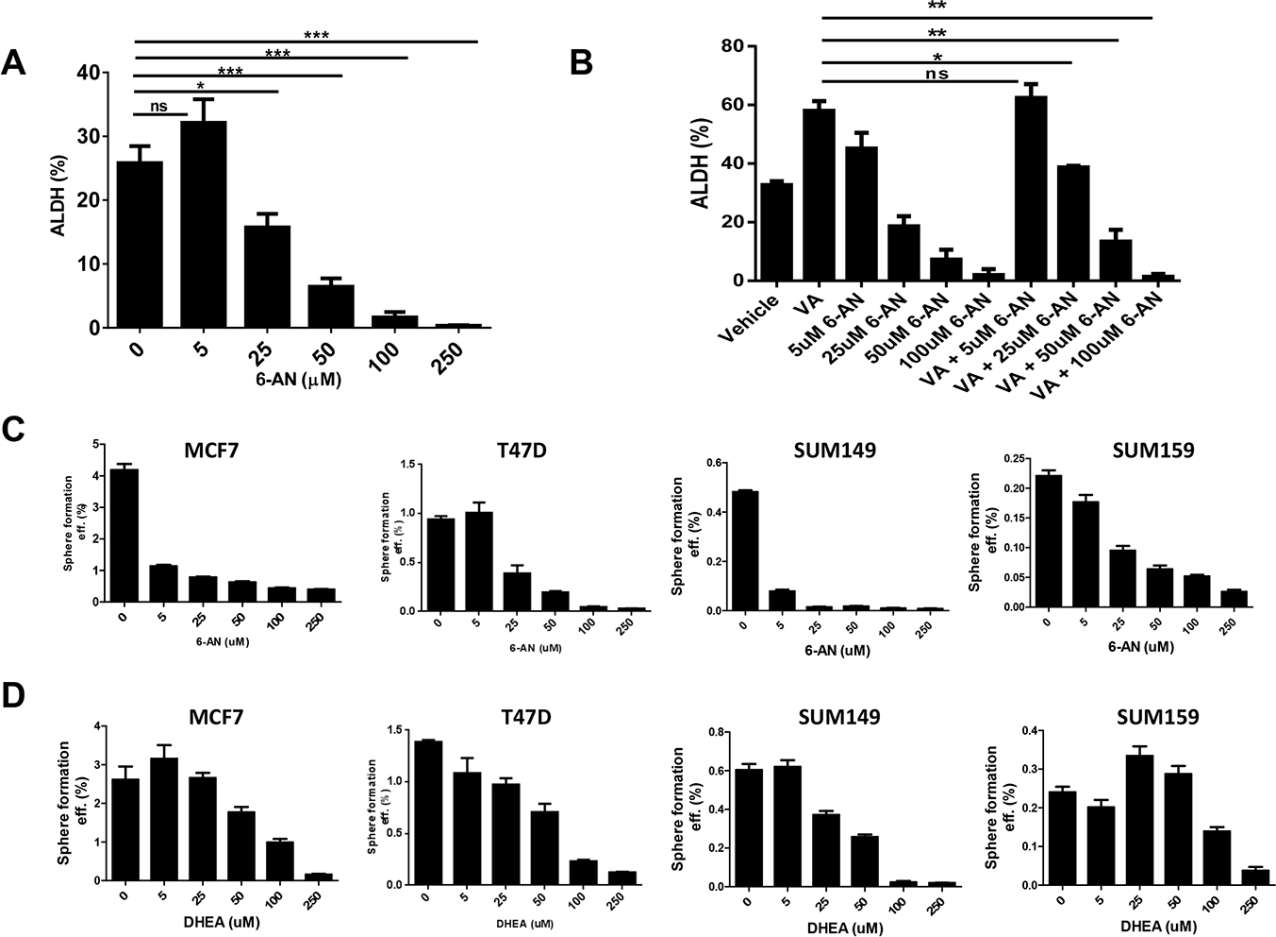
Effect of G6PD inhibitors on stem-like properties of cancer cells (**A**) Flow cytometric analysis to quantify ALDH activity in SUM159 cells. The G6PD/PPP inhibitor 6-aminonicotinamide (6-AN) significantly reduced the percentage of ALDH^+^ cells. (**B**) ALDH^+^ induced by the HDAC inhibitor valproic acid (VA) was reduced significantly by combinatorial treatment with VA and 6-AN. (**C** and **D**) The G6PD/PPP inhibitors 6-AN and dehydroepiandrosterone (DHEA) significantly reduced mammosphere formation efficiency in estrogen receptor-positive (MCF7, T47D) and estrogen receptor-negative breast cancer cell lines (SUM149, SUM159).

Because we found enhanced PPP activity in the HDACi-CSCs, we investigated whether two known inhibitors of the PPP, 6-aminonicotinamide (6-AN) and dehydroepiandrosterone (DHEA), could affect the survival and self-renewal of these stem-like population. We first used an Aldefluor assay to test ALDH activity in SUM159 cells treated with increasing doses of 6-AN and found that 6-AN significantly reduced the percentage of ALDH^+^ cells at concentrations higher than 25 μM (*p* < 0.01, Figure 4A). 6-AN also significantly reduced the proliferation of cells in monolayer culture (Supplementary Figure 3). DHEA also led to a reduction in ALDH^+^ percentage, albeit at a higher concentration (data not shown). We previously showed that the use of anticancer drugs like paclitaxel, erlotinib, and saliomycin did not reduce the HDACi-induced ALDH^+^ subpopulation [17]. However, treatment with 6-AN significantly reduced the ALDH^+^ cells induced by VA (*p* < 0.03, Figure 4B). We also assessed the efficiency of mammosphere formation, another surrogate marker for CSC self-renewal, by treating breast cancer cell lines with inhibitors of G6PD and PPP. We found that mammosphere formation efficiency was significantly reduced in both estrogen receptor positive (MCF7, T47D) and estrogen receptor negative (SUM149, SUM159) breast cancer cell lines, and the effect was more pronounced with 6-AN than with DHEA (Figure 4C, 4D).

**Figure 5:**
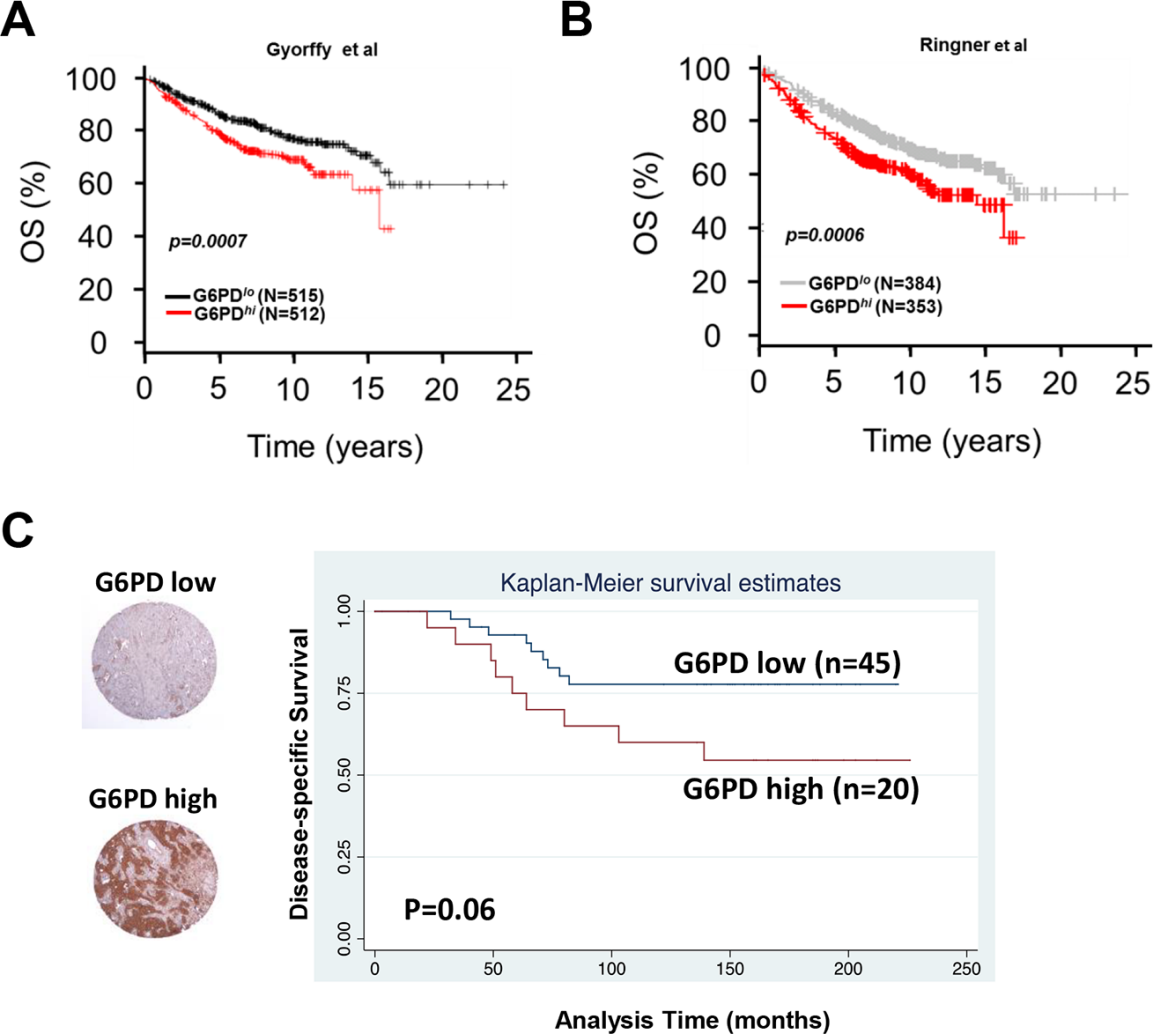
Expression of G6PD in breast cancer patient samples and its correlation with outcome (**A** and **B**) Kaplan-Meier curves depicting high G6PD mRNA expression correlated strongly with reduced overall survival in patients with breast cancer in two publically available databases of breast cancer microarrays. (**C**) Immunohistochemical analysis of G6PD protein in tissue microarrays from patients with lymph node-positive invasive breast carcinoma showed that high G6PD may have been associated with worse disease-specific survival (*p* = 0.06).

We next examined whether G6PD expression correlated with clinical outcomes in patients with breast cancer in publically available datasets and in a tissue microarray from patients with invasive breast cancer. First we used Kaplan–Meier Plotter [19], an online meta-analysis tool for biomarker assessment. Using this tool, we tested whether the mRNA expression of G6PD correlated with overall survival among patients with breast cancer and found that high G6PD mRNA expression correlated strongly with reduced overall survival (*p* = 0.00007, Figure 5A). These findings were validated with another publically available dataset [20] showing poor prognosis for patients with high expression of G6PD mRNA (*p* = 0.00006, Figure 5B). Finally, we assessed G6PD protein expression with immunohistochemical staining of a tissue microarray from patients with lymph node–positive invasive breast carcinoma [21, 22], the median follow-up for whom was 12.5 years. We found a trend toward worse disease-specific survival among breast cancer patients with high G6PD protein levels (*p* = 0.06, Figure 5C), mirroring the outcomes for patients with high G6PD mRNA expression.

## DISCUSSION

We demonstrated that the metabolic properties of stem-like cancer cells induced via HDAC inhibition differ from more differentiated cancer cells and that HDAC inhibitor-induced dedifferentiation promotes reprogramming into the pentose phosphate pathway metabolism. We also showed that PPP inhibitors significantly reduced the stem-like surrogate markers and sensitized HDAC inhibitor induced cancer stem cells, highlighting a potential strategy to preferentially disrupt the metabolism of resistant stem-like cancer cells. Further, by using gene expression data sets and tissue microarray from breast cancer patients, we showed that G6PD was associated with outcomes in patients with breast cancer.

Many solid cancers, including breast cancers, are thought to be organized hierarchically, with a small number of CSCs able to re-grow a tumor although their progeny lack this feature. However, recent studies have challenged this unidirectional hierarchical model of CSCs and shown that even non-tumorigenic differentiated cells can become stem-like cancer cells [23–25]. For example, Meyer et al. demonstrated that noninvasive, epithelial-like CD44+/CD24+ cells can give rise to invasive, mesenchymal CD44^hi^/CD24^lo^ progeny *in vivo* and *in vitro* [23]. Weinberg's group also demonstrated that CSC-like cells can arise spontaneously from more differentiated cell types [24], and Pajonk's group showed that radiation treatment can reprogram breast cancer cells into stem cell-like cells [25]. Most recently, we further demonstrated that differentiated triple-negative breast cancer cells can be reprogrammed to become quiescent resistant stem-like cancer cells through exposure to cancer-targeting agents like HDAC inhibitors [17]. Collectively these studies indicate the complexity and heterogeneity of cancer cells and have important implications for the dynamics of tumor progression and response to therapy.

In heterogeneous populations of cells with different levels of cellular activity, metabolic requirements are likely to vary substantially, which in turn could result in different responses to metabolic targeting. Gaining further insights into the metabolic properties of CSCs may reveal physiological dependencies that can be targeted for therapy. Several recent studies have investigated the metabolic state of CSCs and their differentiated progeny, although the findings from these studies are inconclusive. Some have shown that CSCs are more dependent on oxidative phosphorylation than are differentiated cancer cells [11–13]. Vlashi and colleagues reported that breast CSCs rely on oxidative phosphorylation, but their more differentiated progeny has a more glycolytic phenotype [13]. An earlier study from the same group also reported comparable findings in glioma CSCs [11]. Likewise, Jordan's group found that quiescent leukemia stem cells from patient-derived samples also relied primarily on oxidative phosphorylation [12]. Conversely, others have demonstrated that CSCs rely predominantly on glycolytic metabolism. Feng et al. found that breast CSCs in mouse and human tumors have a more glycolytic phenotype than do their differentiated progeny [15]. Others reports also showed a shift from mitochondrial oxidative phosphorylation towards glycolysis in breast and nasopharyngeal CSCs [14, 16]. In the current study, we observed metabolic reprogramming in HDAC inhibitor-induced, quiescent CSC-like cells that contrasts with that of the un-induced, differentiated cancer cells, with activation of the glycolytic pathway accompanied by high PPP activity, as indicated by high glucose consumption, high NADPH production, and high G6PD expression. Similar observations were made with fibroblasts induced into quiescence, which were found to have a high metabolic rate, including high rates of glycolysis and high PPP activity [26]. However, unlike previous findings that CSCs have low ROS levels [27, 28], our work here showed that HDACi-CSCs had higher ROS levels than did the differentiated cancer cells, despite increased production of NADPH, a known intracellular ROS-detoxifying molecule. This effect may have resulted from one or both of the direct effects of HDAC inhibitors on activation of G6PD [29] and increased generation of ROS [30, 31]. We further found that ROS scavengers such as SOD2 were reduced in the HDACi-CSCs relative to the control cells, which may have contributed to the high ROS levels in the CSC-like cells. Moreover, PPP may have been increased in response to the oxidative stress from HDAC inhibitors, which elicits high ROS levels and provokes an adaptive response by augmenting the PPP. It is also possible that the NADPH created may help the HDAC inhibitor-treated, quiescent and cells to synthesize lipids in addition to detoxifying free radicals, as has been reported in a previous study [26] that showed quiescent fibroblasts diverted glucose to the pentose phosphate pathway which was directed in part toward the synthesis of fatty acids.

The PPP has a pivotal role in helping glycolytic cancer cells to meet their anabolic demands and combat oxidative stress [18]. The PPP is regulated oncogenically by numerous pro-oncogenic signaling pathways such as P53, p73, PI3K/AKT, and Ras [32–35]. Jiang et al. demonstrated that p53 inhibits the PPP through directly inactivating G6PD enzyme activity [32]. Another study found that the p53-related protein p73 induces the expression of G6PD, enhances the PPP, and promotes proliferation of cancer cells [33]. Studies of the metabolic consequences of K-Ras activation in a mouse model of pancreatic cancer revealed that it serves a vital role in controlling tumor metabolism through stimulating glucose uptake and channeling glucose intermediates into the PPP [35]. These studies have shown that dysregulation of the PPP flux significantly affects cancer growth and survival, making the PPP an interesting target in tumor cells.

In addition to regulating the proliferation and survival of cancer cells, the PPP has been implicated in promoting resistance to cancer therapies [36–38]. We recently showed that HDACi-CSCs are highly resistant to chemotherapeutic agents such as paclitaxel, salinomycin, erlotinib, and other agents [17]. In the current study, we demonstrated that the stem-like cells are sensitive to PPP inhibitors, particularly 6-AN, which not only reduced the ALDH^+^ subpopulation and mammosphere formation efficiency but also significantly reduced the VA-induced ALDH^+^ population. Further functional endpoint studies with VA and 6-AN treatment of tumor xenografts would serve to validate the *in vitro* findings. Because of the neurotoxic effects of 6-AN and the lack of specificity of DHEA, new inhibitors to modulate the PPP pathways are being developed, including two natural products, catechin gallates [39] and rosmarinic acid [40], that were recently discovered to inhibit G6PD. Such drugs that modulate the PPP could be used as potential tools in tumor therapy. Given the beneficial effects of HDAC inhibition on bulk tumor cells, combination therapies with HDAC inhibition and G6PD inhibitors in patients with high-G6PD-expressing tumors is an attractive combination for future study.

## MATERIALS AND METHODS

### Cell culture and reagents

The breast cancer cell lines SUM149 and SUM159 were obtained from Asterand (Detroit, MI). Both cell lines were cultured in Ham's F-12 medium supplemented with 10% fetal bovine serum (FBS), 1 μg/mL hydrocortisone, and 5 μg/mL insulin. T47D and MCF7 cells were obtained from the American Type Culture Collection (Manassas, VA, USA) and cultured in DMEM/F12 supplemented with 10% FBS (T47D) or MEM supplemented with 10% FBS, 0.1 mM nonessential amino acids, 1 mM sodium pyruvate, 1 μg/mL hydrocortisone, and 5 μg/mL insulin (MCF7). The following reagents were used at the doses indicated and as described in the text and figure legends: VA (Sigma), SAHA (Cayman), 6-AN (Cayman), and dehydroepiandrosterone (DHEA; Cayman).

### Aldefluor assay and sorting of ALDH negative/positive population

The Aldefluor assay and sorting of the ALDH negative/positive cell populations were described in our previous study [17]. Briefly, about 5 × 10^5^ cells were suspended in Aldefluor assay buffer containing ALDH substrate and incubated for 30 min at 37°C. As a negative control for each sample, cells were incubated with 50 mmol/L of the specific ALDH inhibitor diethylaminobenzaldehyde (DEAB). Aldefluor fluorescence was excited at 488 nm and fluorescence emission was detected by using a Beckman Coulter machine. Data files were analyzed with FlowJo software (Treestar, Ashland, OR). For sorting, gates were established using ALDH–stained cells treated with DEAB as negative controls and taking the high negative and positive cells. Sorted Aldefluor-negative (ALDH^−^) and Aldefluor-positive (ALDH^+^) cells were treated with 1 mM VA, 1 μM SAHA or vehicle control for 7 days for further experiments.

### MTS assay

ALDH^−^ and ALDH^+^ cells were plated in 96-well plates (5,000 per well) and treated with 1 mM VA, 1 μM SAHA or vehicle control for 7 days. Following treatment, the culture medium was then replaced with fresh medium mixed with 20% MTS [3-(4,5-dimethylthiazol-2-yl)-5-(3-carboxymethoxyphenyl)-2-(4-sulfophenyl)-2H-tetrazolium, inner salt] (Promega, Madison, WI). After 2 hours, the absorbance at 490 nm was determined by using Victor X3 plate reader (PerkinElmer, Waltham, MA). The values obtained were normalized to the number of cells in each well.

### Glucose uptake and lactate production assays

The fluorescent 2-deoxyglucose analog 2-[N-(7-nitrobenz-2-oxa-1,3-diazol-4-yl)amino]-2-deoxy-D-glucose (2-NBDG; Invitrogen) was used to measure glucose uptake. Sorted ALDH^−^ and ALDH^+^ cells were cultured in 6-well plates for 7 days in the presence or absence of HDAC inhibitors, after which medium was removed and the cells were incubated with 10 μM 2-NBDG for 1 h, washed twice, and analyzed by flow cytometry at an excitation wavelength of 488 nm. The mean fluorescence intensity (MFI) served as a measure for 2-NBDG uptake on a per-cell basis.

To assess lactate production, cells were sorted and treated in a similar manner and the amount of lactate present in the medium was then estimated with a Lactate colorimetric Assay Kit (BioVision Research Products). Medium was collected and diluted 1:10 in lactate assay buffer according to the manufacturer's instructions, and the absorbance at 570 nm was determined by using a Victor X3 plate reader (PerkinElmer). The amount of lactate produced by the cells in each sample was calculated by subtracting the amount of lactate in the medium (without cells) from the amount of lactate in the medium from each sample. The values obtained were normalized to the number of cells in each well.

### Measurement of NADP+/NADPH levels

The NADP^+^/NADPH ratio was determined with an NADP/NADPH quantification colorimetric kit (Biovision, USA). Briefly, cells were washed with phosphate-buffered saline, scraped, collected, and centrifuged, after which 10^6^ cells in each group were resuspended in the NADP^+^/NADPH extraction buffer included in the NADP^+^/NADPH quantification kit. The NADP^+^/NADPH ratio was determined according to the manufacturer's protocol. Absorbance at 450 nm was measured with a Victor X3 plate reader (PerkinElmer).

### Measurement of ROS levels

Sorted ALDH^−^ and ALDH^+^ cells were cultured in 6-well plates and treated with indicated doses of HDAC inhibitors or vehicle control for 7 days. At that time medium was removed and cells were incubated with 10 μM H2DCF-DA (Invitrogen) in medium for 1 hour in the dark. After DCF-DA staining, cells were trypsinized, washed twice and resuspended in phosphate-buffered saline, and then analyzed by flow cytometry with a FACScalibur (BD Bioscience, San Jose, CA).

To measure superoxide, we used Mitosox Red (Invitrogen, CA, USA), a fluorescent probe targeted to mitochondria. Briefly, sorted ALDH^−^ and ALDH^+^ cells were cultured in 6-well plates and treated with indicated doses of HDAC inhibitors or vehicle control for 7 days. Cells were then incubated with 10 μM MitoSOX Red reagent for 15 min at 37°C. Cells were then washed twice and resuspended in phosphate-buffered saline and then analyzed by flow cytometry on a FACScalibur (BD Bioscience), with the excitation set at 510 nm. The mean fluorescence intensity (MFI) served as a measure for the DCFDA and Mitosox Red levels on a per-cell basis.

### Western blotting

Sorted ALDH^−^ and ALDH^+^ cells were treated with HDAC inhibitors or vehicle control for 7 days, followed by lysis in 1× radio-immunoprecipitation assay lysis buffer containing 1 μM phenylmethylsulfonyl fluoride and 40 μg protein and subjected to electrophoresis on 4%–20% gradient SDS-polyacrylamide gels (Invitrogen). Membranes were then incubated with anti-G6PD antibody (Abcam). GAPDH antibody was used as a loading control.

### Mammosphere formation efficiency

Cancer stem/progenitor cells can be enriched by propagating cells in serum-free, growth factor-enriched conditions; when the cells come from breast cancer, they form mammospheres [3]. To generate primary mammospheres, treated and untreated cells were grown in serum-free, growth factor–enriched medium in low attachment plates as we described elsewhere [4, 17]. Briefly, 2 × 10^4^ cells/mL were cultured for 5–7 days in serum-free MEM supplemented with 20 ng/mL basic fibroblast growth factor, 20 ng/mL epidermal growth factor, and B27 (all from Invitrogen) in 6-well ultra-low attachment plates. The mammosphere medium did not contain HDAC inhibitors throughout the duration of the mammosphere formation assays.

### Data mining

The expression of G6PD mRNA was assessed in samples from two public databases of different cohorts of patients with breast cancer, the Kaplan-Meier Plotter [19] and Gene Expression-Based Outcome for Breast Cancer Online (GOBO) [20]. Survival information for the patients supplying the samples was evaluated in both databases, in which patients had been stratified into groups of high and low G6PD expression by using a database-selected “cutoff” point.

### Patient characteristics, tissue microarray, and immunohistochemical staining

The tissue microarray has been described previously [21, 22]. Briefly, 94 patients who had undergone surgery at The University of Texas MD Anderson Cancer Center between 1986 and 1994 were included. Inclusion criteria were resectable stage II or IIIA breast cancer with axillary lymph node metastases, surgical treatment with mastectomy and axillary dissection without irradiation, age younger than 75 years at diagnosis, no evidence of distant disease at diagnosis, and no prior or concurrent malignancy. Tissue microarrays were prepared from the paraffin blocks of the primary breast tumors by using a manual tissue puncher or array (Beecher Instruments). Up to 3 cores, each 0.6-mm in diameter, were cut from each primary tumor. Immunohistochemical staining of the microarrays was done at the Research Histopathology Facility Core Laboratory at MD Anderson with a polyclonal rabbit anti-G6PD antibody (ab993, Abcam). Staining was scored without knowledge of the clinical outcome. Staining for G6PD was scored for invasive tumor cells only. Any nuclear and/or cytoplasmic staining was scored as weak, moderate, or strong, and the percentage of invasive tumor cells with staining was scored as 25% or less, 26%–50%, 51%–75%, or > 75%. Tumors with strong intensity staining in greater than 50% were considered to have “high expression”, and those with weak to moderate intensity staining in 50% or less were considered to have “low expression”. A log–rank test was used for statistical analysis.

### Statistical analysis

Statistical analyses were done with GraphPad Prism version 6. All data are represented graphically as means ± SEM. *P* values < 0.05 in paired two-sided tests were considered to indicate statistical significance. The Kaplan-Meier method was used to evaluate disease-specific survival according to G6PD protein expression, measured by immunohistochemical staining.

## SUPPLEMENTARY MATERIALS FIGURES


