# Supplementary material for: Histone deacetylase inhibitor-induced cancer stem cells exhibit high pentose phosphate pathway metabolism
Source: Oncotarget. 2016 Apr 7;7(19):28329–39. doi: 10.18632/oncotarget.8631 (PMC5053589; doi:10.18632/oncotarget.8631)
Supplement: Supplementary file 1 [file oncotarget-07-28329-s001.pdf]

Histone deacetylase inhibitor–induced cancer stem cells exhibit high pentose phosphate pathway metabolism

Supplementary Materials

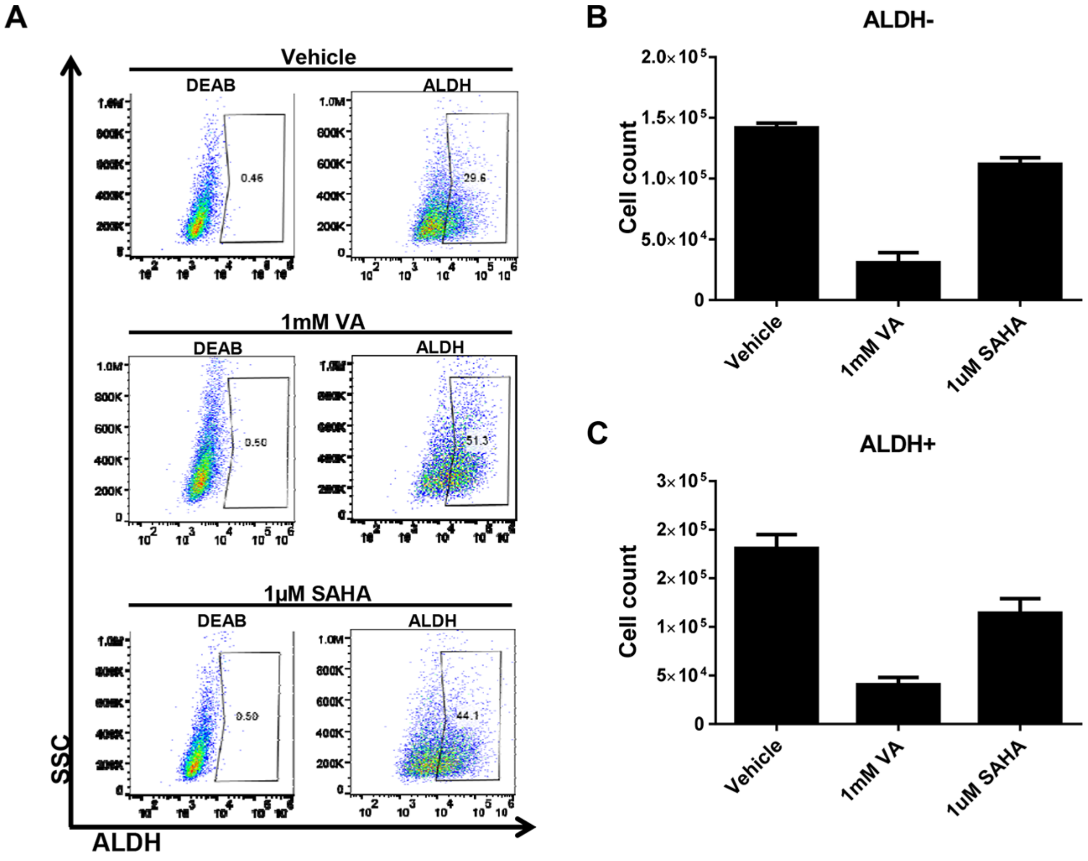

Supplementary Figure S1: Effect of HDAC inhibitors on CSCs and proliferation. (**A**) A representative flow cytometry diagram illustrating increased ALDH$^+$ cells in unsorted SUM159 cells treated with HDAC inhibitors. Gating is set to DEAB-inhibitor control cells for each sample. (**B** and **C**) Cell viability in ALDH$^-$ (B) and ALDH$^+$ (C) cells treated for 7 days showed reduced viability with HDAC inhibitor treatment.

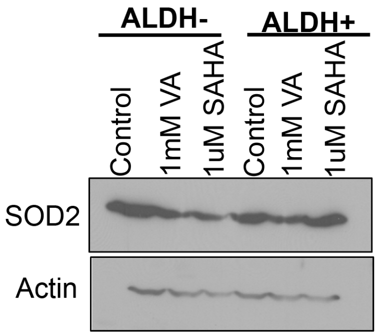

Supplementary Figure S2: SOD2 expression in HDACi-CSCs. Immunoblotting showed a reduction in SOD2 protein expression in ALDH⁻ and ALDH⁺ cells treated with HDAC inhibitors, particularly VA.

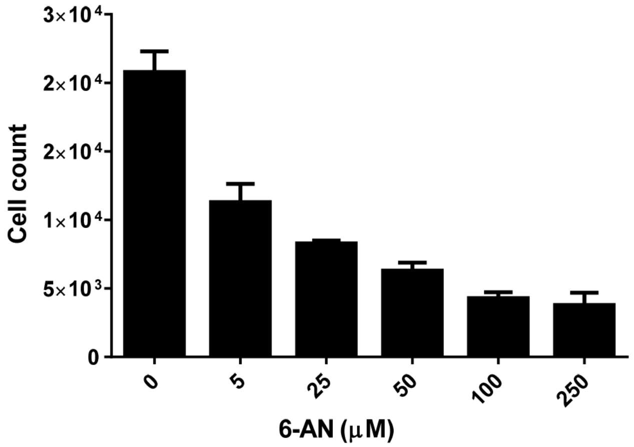

Supplementary Figure S3: The effect of 6-AN on cell viability. ALDH⁻ cells showed reduced viability with increasing doses of 6-AN.
